# Ultrastructure of primary pacemaking cells in rabbit sino‐atrial node cells indicates limited sarcoplasmic reticulum content

**DOI:** 10.1096/fba.2018-00079

**Published:** 2020-01-07

**Authors:** Ramesh Iyer, Oliver Monfredi, Manuela Lavorato, Mark Terasaki, Clara Franzini‐Armstrong

**Affiliations:** ^1^ Division of Cardiology Children Hospital of Philadelphia Philadelphia PA USA; ^2^ Laboratory of Cardiovascular Sciences NIA IRP NIH Baltimore MD USA; ^3^ The Johns Hopkins Hospital Department of Cardiology Baltimore MD USA; ^4^ Department of Cell and Developmental Biology University of Pennsylvania Philadelphia PA USA; ^5^ Department of Cell Biology University of Connecticut Health Center Farmington CT USA

**Keywords:** gap junctions, pacemaker cells, sarcoplasmic reticulum, sino‐atrial node

## Abstract

The main mammalian heart pacemakers are spindle‐shaped cells compressed into tangles within protective layers of collagen in the sino‐atrial node (SAN). Two cell types, “dark” and “light,” differ on their high or low content of intermediate filaments, but share scarcity of myofibrils and a high content of glycogen. Sarcoplasmic reticulum (SR) is scarce. The free SR (fSR) occupies 0.04% of the cell volume within ~0.4 µm wide peripheral band. The junctional SR (jSR), constituting peripheral couplings (PCs), occupies 0.03% of the cell volume. Total fSR + jSR volume is 0.07% of cell volume, lower than the SR content of ventricular myocytes. The average distance between PCs is 7.6 µm along the periphery. On the average, 30% of the SAN cells surfaces is in close proximity to others. Identifiable gap junctions are extremely rare, but small sites of close membrane‐to‐membrane contacts are observed. Possibly communication occurs via these very small sites of contact if conducting channels (connexons) are located within them. There is no obvious anatomical detail that might support ephaptic coupling. These observations have implications for understanding of SAN cell physiology, and require incorporation into biophysically detailed models of SAN cell behavior that currently do not include such features.

**Abbreviations**
Free SR: junctional SR
PC: peripheral coupling
RyR: Ryanodine receptor
SAN: Sino‐atrial node
SR: sarcoplasmic reticulum

## 1 INTRODUCTION

The pacemaking rhythm that controls the overall beating rate of the heart in health originates in the sino‐atrial node (SAN). The SAN is comprised of an anatomically and functionally heterogeneous collection of cells all capable of spontaneously and rhythmically generating action potentials, demonstrating the key property of automaticity.^1^, ^2^ Among these, the most highly specialized SAN cells are the leading or dominant pacemakers, those with the fastest rate of diastolic depolarization under a given set of physiological parameters. They are located in the central region of the SAN and are the least anatomically developed cells, with the lowest density of organelles, particularly myofibrils.^3^ As one moves away from this central region, there is a transition in the properties of spontaneous action potentials produced by the cells and in their structure, with the addition of myofibrils, an increase in SR, and the presence of internal corbular SR. The significance of the structural transition in functional terms and the question of whether true atrial cells are infiltrated in the pacemaking core of the node have been variably interpreted. On the one hand, the structural transition is mostly described as a gradation of myofibrillar content from center to periphery of node, which has been correlated with variations in electrophysiological parameters, the so‐called “gradient model”.^3^, ^4^ Other investigators^5^ find that cells with typical characteristics of atrial myocytes are found interspersed within the inner core of the SAN, and propose that a gradual increase in the density of infiltrating atrial‐type cells is at the basis of the transition from nodal to atrial electrical properties, the so‐called “mosaic model.” Regardless of whether the node contains a gradual local variation of cells or is constituted of a mosaic of mixed cells, the heterogeneity has a meaning in terms of dependable function of the SA node as a pacemaker.^1^

Two main (not necessarily mutually exclusive) schools of thought have dominated debate on the origin of the spontaneous pacemaker potential in SAN cells.^6^ In the first, functional parameters of plasmalemmal ionic channels are considered fully responsible for the slow depolarization and the derived action potential when threshold is reached. The discovery of the *funny* current specific to SAN cells^7^ and the further characterization of HCN4 (hyperpolarization‐activated, cyclic‐nucleotide gated four) as the major carrier of the *funny* current^8^ laid a strong foundation for the ionic basis of the intrinsic rhythmicity. An alternate proposal is that rhythmicity is regulated by calcium transients via voltage‐gated sarcolemmal Ca^2+^ channels, SR calcium stores, and the Na^+^/Ca^2+^ exchanger.^9^ This proposes that an exponential increase in NCX current at end‐diastole, due to spontaneously propagated local SR calcium release, affects SAN pacemaking frequency.^10^ Since the discovery that internal calcium delivery in these cells of small size could drive depolarization (11 see 6 for a review), the magnitude of this effect in driving physiological pacemaking has been hotly debated.^12^ The current paradigm suggests that the two mechanisms function in concert, as a coupled clock system that is mutually entrainable, robust, and reliable.^10^

The question of how SAN cells communicate with each other and with the atrial myocytes that surround them to ensure regular, reliable conduction of the impulse within the SAN and out of it provides an interesting puzzle. On the one hand, the cells of the major pacemaking core must communicate between themselves and either with the surrounding cells that, in turn, mediate access to the atrial cells or with atrial cells that may have infiltrated the node.^5^ On the other hand, the primary pacemaking cells must be protected from retrograde transmission that would overcome their rhythmic signal. How this is achieved is not clear. Immunolabeling experiments (summarized in 13) have been hard to interpret. Labeling for the most abundant connexon in heart (CX43) is mostly negative,^14^ but different isoforms may be involved. Verheijck et al^15^ show very clear punctate anti‐Cx45‐positive sites in nodal area of the mouse, and antibodies against CX40 are positive for some cells, but can also be totally negative for relatively large groups of them. Masson‐Pevet, using electron microscopy, showed the images of small “classical” gap junctions with a number of connexons forming tight clusters (quoted in Ref. 13, see Ref. 3, 16, 17), but did not indicate whether these were found in the SAN cells of the inner core. Other researchers have also found such small gap junctions, although quite rarely.^18^ Finally, the suggestion was made that very small punctate connections may be the preferred site of intercellular communication by providing for the location of small clusters of conductive connexons.^19^ The more recently proposed mechanism of ephaptic coupling has not been explored in the case of the SA node. It will be dealt with in the discussion section. The aim of this investigation is to provide an in‐depth ultrastructural description of SAN cells from the central region of the rabbit SAN. The study is restricted to the cells constituting the main pacemaking region and it provides a quantitation of the SR elements that should be taken into consideration in establishing the relative importance of the calcium‐driven internal oscillator in driving pacemaker activity. It turns out that the cells have much smaller SR components than previously assumed, certainly when compared to ventricular myocytes, so initial modeling based on data from ventricle may need to be reconsidered for these SAN cells.

## 2 MATERIALS AND METHODS

**Figure 1:**
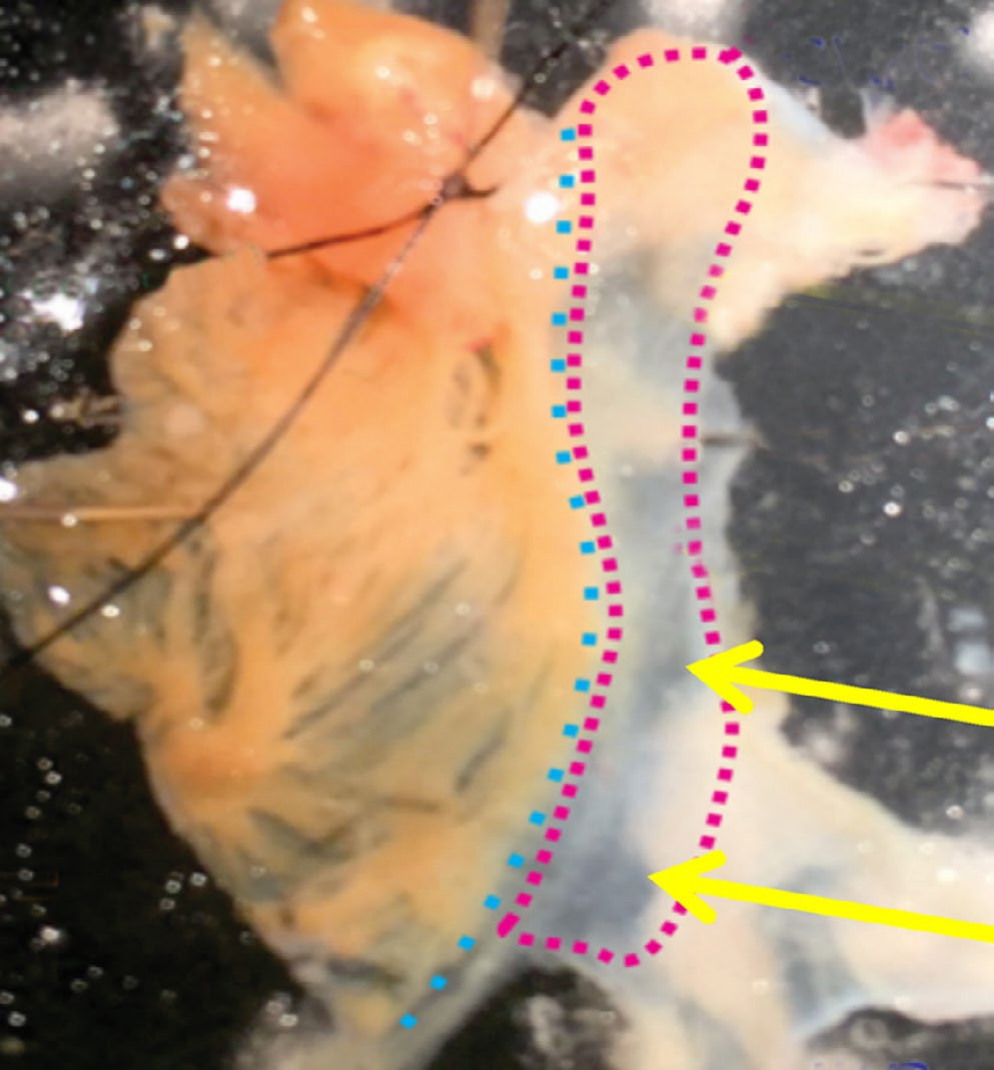
The SA Node. Dissection of a SAN from rabbit heart previously fixed by perfusion. The sample is pinned, the ligation at the upper left was used for help in the dissection. The magenta dotted line follows the outline of the SAN upper (top) and lower regions; the cyan dots follow the crista terminalis; the yellow arrows point to two of the almost transparent regions that were embedded and sectioned for EM. Atrial tissue is at the left of the SAN

Sinus nodes were isolated from adult male New Zealand White rabbits in accordance with the National Institutes of Health Guidelines for the Care and Use of Animals (Protocol No. 034‐LCS‐2019). New Zealand White rabbits (Charles River Laboratories) weighing 1.8‐2.5 kg were deeply anesthetized with pentobarbital sodium (50‐90 mg/kg). The heart was removed quickly and placed in solution containing the following (in mM): 130 NaCl, 24 NaHCO_3_, 1.2 NaH_2_PO_4_, 1.0 MgCl_2_, 1.8 CaCl_2_, 4.0 KCl, and 5.6 glucose equilibrated with 95% O_2_‐5% CO_2_ (pH 7.4 at 35.5°C). Excised hearts were initially retrogradely perfused by gravity with heparinized Tyrode solution, followed by 75 mL of 3% glutaraldehyde 0.1M cacodylate buffer pH 7.2. After a short period of time, the right atrium and associated sinus node were dissected out and kept in the fixative for a variable period of time at 4°C, up to several days. The node region was pinned (Figure 1) and the central partially translucent areas (arrows) where the leading pacemaker site exists under baseline conditions were identified and further dissected out. The tissue was rinsed in cacodylate buffer, and either postfixed in 2% OsO_4_ in the same buffer containing 0.6% K_3_Fe(CN)_6_, or postfixed in 2% OsO4 in the same buffer for 1 hour at room temperature, rinsed in H_2_O and en‐bloc stained in aqueous saturated uranyl acetate for 1 hour.^20^ The tissue was dehydrated in ethanol and acetone and embedded in Epon. Thin (50‐60 nm) sections were cut at right angles to the thin layer of the SA node on a Leica Sitte microtome and stained with “Sato” lead citrate.^21^ Sections were imaged at 60‐80KV either in a Phillips 410 (Mahwah. NJ) or in a JEOL 1010 (JEOL USA) electron microscopes, both equipped with a Hamamatsu camera (Advanced Microscopy Techniques, Chazy, NY). Average quantitative information was obtained through an appropriate morphometric analysis of a number of thin section images as described by Weibel et al^21^ Measurements were taken on digitized images using the freely available NIH Image J program.

## 3 RESULTS

### 3.1 SAN architecture and cell identification

**Figure 2:**
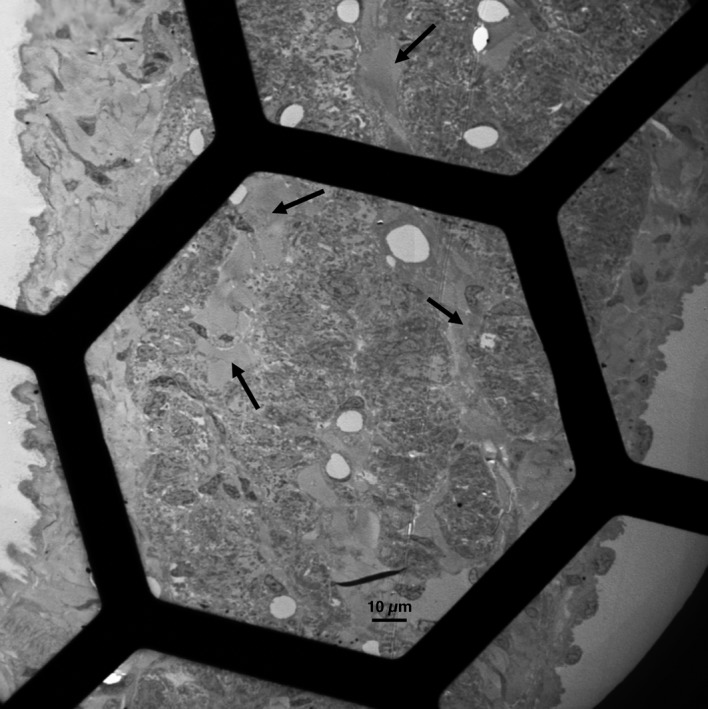
Low‐power image of a section across the center of a node area such as indicated by arrows in Figure 1. Dense collagen bands (arrows) separate cell‐rich bands which are also infiltrated by collagen bundles. Connective tissue plus endothelium (right) and mesothelium (left) cover the two surfaces. No obvious ultrastructural differences were observed between cells on the two sides

**Figure 3:**
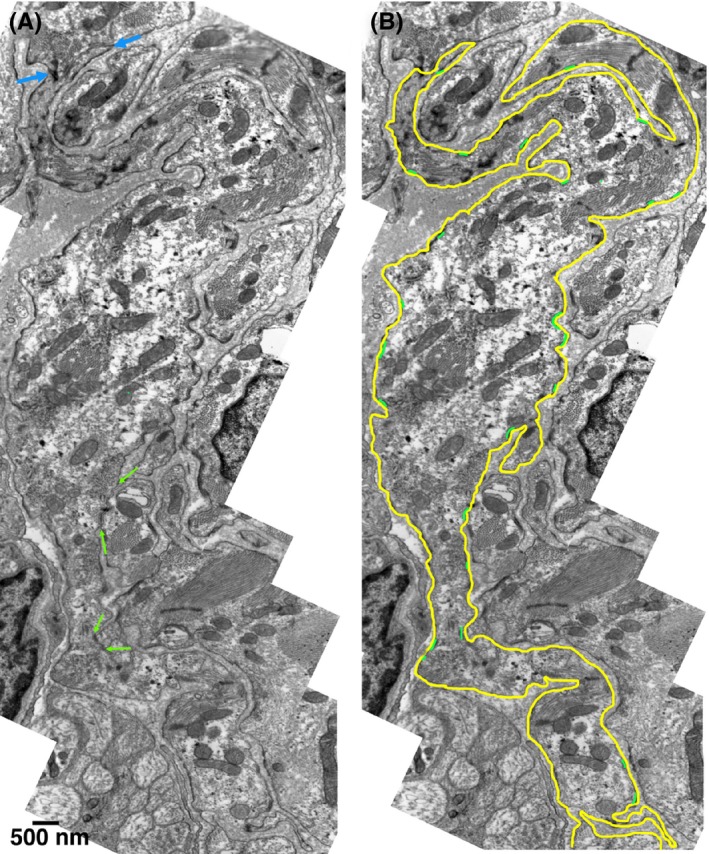
A, This image is constructed from a series of low magnification micrographs: occasional discontinuities occur where the matching was not perfect. The outline of an elongated cell is shown in its entirety. Most of the cell surface is isolated from neighboring cell by either a single of a double layer of basal lamina, but close contacts are often present (between green arrows). At the top of the image, the cell ends into a junction connecting the two cells via small actin‐based adhering junctions, such as found at intercalated discs of the working heart (between blue arrows). B, Same cell as in (A). The rough yellow outline enhances the very long and thin shape of the cell, with a branch at the upper end, as shown in the literature for isolated cell. Short green segments indicate the position and approximate length of peripheral couplings

The translucent region of the node is composed of layers of dense collagen bands that separate strands of pacemaking cells (Figure 2). The epicardial and endocardial surfaces of the thin node are easily identified based on details of the epithelium and connective tissue covering them. The cells located in the central portion of the node have been previously identified as the primary pacemakers.^1^ Anatomically, these are the least developed cells, containing the lowest density of organelles, particularly myofibrils. The cells are folded up and closely spaced, so each cell has extensive proximity to several other cells. Figure 3A,B shows the outline of a cell that was followed in its entirety within the section. The overall shape is quite similar to that described for isolated cells—the cell is long and thin, and in this case, it has a bifurcation at one end, as shown by Verheijck et al.^5^ In this image, the cell ends in a junction that connects it to the adjacent cell via small actin‐based adhering junctions, such as those found in the intercalated discs of the working myocardium (between blue arrows). Close contacts with two different cells are made along the lateral borders (green arrows). Green marks indicate the presence and approximate size of jSR peripheral couplings. Figures S1 and S2 show the closely apposed outlines of two cells reconstructed from serial sections imaged in SEM (see methods). Note that both cells have a quite convoluted shape and face each other over most of their surfaces.

**Figure 4:**
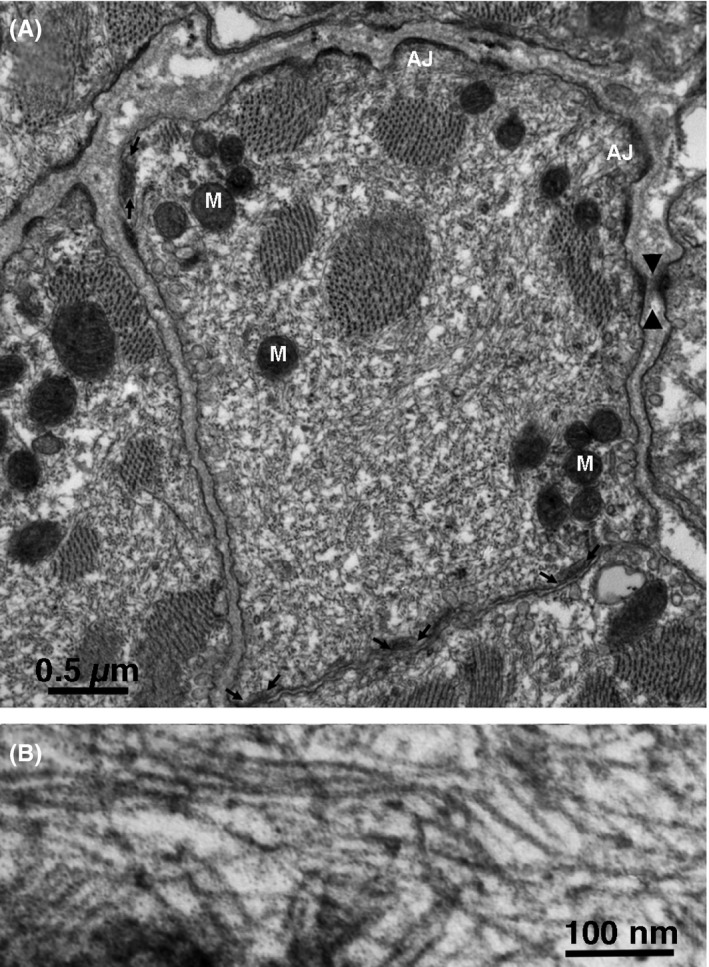
A, Profile of a typical “dark “cell. The cytoplasm is tightly packed with intermediate filaments running in all directions in addition to few mitochondria (M, small, round objects) and scarce myofibrils. Peripheral couplings are present (between small arrows) but free sarcoplasmic reticulum is rare. On the upper edge, the two adjacent cells are separated by their own independent basal laminae; on the left side, the two cells share a common basal lamina; along the lower edge, the two facing cells are closely apposed, with a separation of ~100 nm. Several sites along the perimeter show densities associated with adhering junctions (AJ). Most of them (at the cell upper edge) allow anchoring to the basal lamina, while occasionally a corresponding structure in an apposed cell established a cell‐to‐cell anchorage (arrow heads). B, In another dark cell, the intermediate filaments are clearly visible because they are mostly parallel to the plane of the section

**Figure 5:**
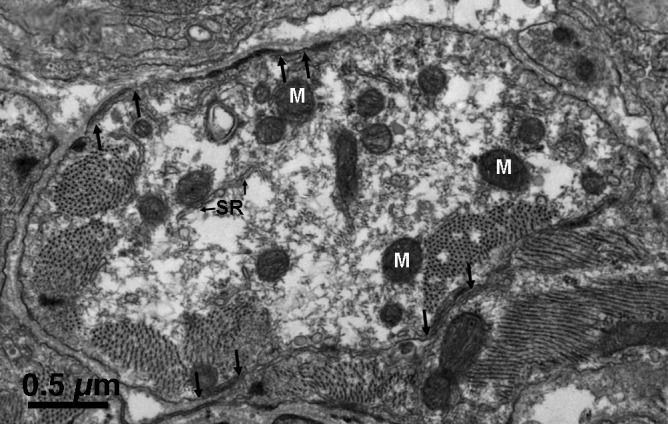
A “pale” cell profile shows large apparently empty areas and few intermediate filaments. It has the same content of peripheral couplings (between arrows) and mitochondria (M) as dark cells, very little internal free SR (SR, small arrows) and varied relationship with other cells along its border. In this image, the entire lower region of the cell closely faces a neighboring one. Infrequent views of cells that are included in their entirety within in the section plane (eg, Figure 3) show that the “light” appearance with many apparently empty spaces is maintained over the whole visible region of the cell. We conclude that “light” and “dark” cell profiles do not belong to the same cell

**Figure 6:**
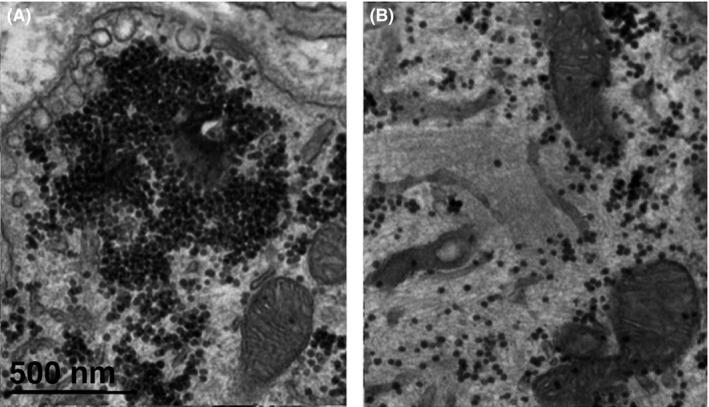
Both light and dark cells are highly endowed with glycogen. Unlike the rest of the samples, this tissue was treated with potassium ferrocyanide (see methods and 1, 23, 30) that allows visibility of glycogen granules. In A (pale cell), glycogen granules are clumped in “empty spaces,” while in B (dark cells) the glycogen granules are dispersed between neurofilaments

In most thin section images, individual cells appear as short profiles that vary widely in appearance and size because the cells are cut at odd angles relative to their long axis (Figures 4, 5, 6). For brevity of description, we use the term “cell” in reference to the randomly sectioned cell profiles, although usually they represent only a small portion of the actual cell. In the literature, “dark” and “pale” cells have been described, based on their density in light microscope images. Higher magnification electron micrographs reveal that the difference is due to the content of intermediate filaments, also known as neurofilaments, in the cytoplasm. Figure 4 illustrates two typical “dark “cells, with cytoplasm completely filled by a dense network of filaments sectioned at varying angles (Figure 4A,B). Noteworthy details of the cell in Figure 4 are as follows: scarce myofibrils, few mitochondria, several peripheral couplings (between arrows), but extremely few (or none) membrane‐limited profiles of free SR. A typical “pale” cell (Figure 5) is characterized by apparently empty areas of various sizes, interspersed with a scarce content of cytoskeleton, including scanty neurofilaments. Other details are similar to those already described for the dark cell: few myofibrils and mitochondria, peripheral couplings (between arrows), and some adhering junctions for anchorage to the basal lamina. Cell with a content of neurofilaments intermediate between the dark and light are rare. A few tubular elements with smooth surface membranes, presumably free SR, are visible. The striking difference between the profiles in Figures 4 and 5 is not due to variations in intermediate filament content along a single cell because images such as the one in Figure 3 show that the intermediate filament content does not vary along the length of the cell. We conclude that the pale and dark profiles represent different types of cells. The distinction is not binary since some cells have an intermediate content of neurofilaments.

Both dark and pale cells are extremely rich in glycogen, as demonstrated after cells are treated with potassium ferrocyanide rather than uranyl acetate to increase the contrast (Figure 6, see methods). Glycogen‐protein “granules” of uniform size^23^ accumulate in large clumps, filling the previously apparently empty areas of the pale cells (Figure 6A) and are dispersed in small groups between the intermediate filaments of the dark cells Figure 6B). So, the apparently empty appearance of the light cells is due to the clustering of glycogen granules into large lumps.

### 3.2 Quantitative data on SR content and distribution

**Figure 7:**
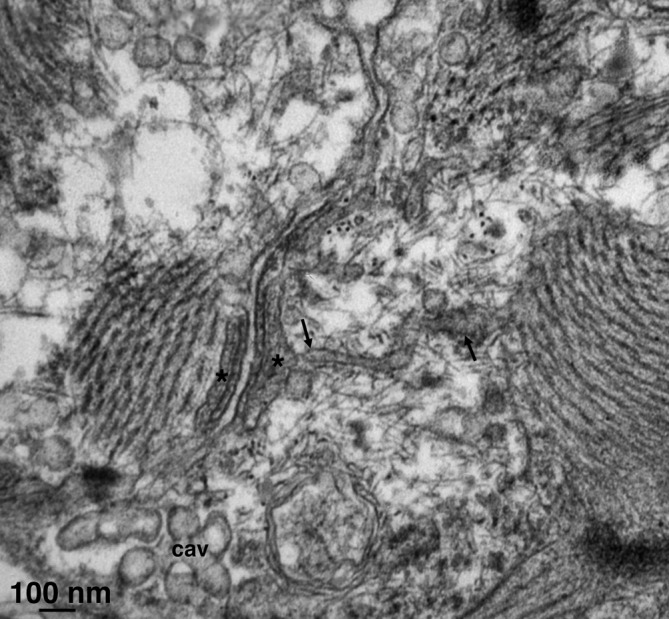
The jSR forms peripheral couplings at the cell edge (asterisks), connected to the surface membrane by RyRs, visible as small periodic elements in the junctional gap between SR membrane and the plasmalemma. Occasionally, peripheral couplings in two adjacent cells face each other as is the case here. Arrows point to free SR. Cav indicates a group of caveolae

All cells have a relatively high frequency of peripheral couplings, formed by associations of small flat junctional SR cisternae with the plasmalemma via visible arrays of feet (RyRs) (Figure 7). A count of the frequency of PCs along the sectioned profiles of plasmalemma shows an average of 5.3 PCs over an average perimeter length of 40.4 µm for the same cell profiles (from 30 profiles), indicating an average frequency of 0.13 PC/µm of perimeter or a calculated average inter PC distance along the perimeter of 7.6 µm. Note that the average measurements take into account domains with a higher PC frequency as well as areas that have far fewer PCs. The overall shape of an entire cell in Figure 3 clearly shows that PC positioning varies along the cells. Additionally, due to surface membrane convolutions, the distances along the plasmalemma are larger than the spacings along straight lines. It is not clear why a considerably lower frequency was estimated by Masson‐Pevet et al,^17^ who quoted sub‐micrometer distances between RyR clusters. There are no T tubules; therefore, no dyads and also corbular SR is absent.

**Figure 8:**
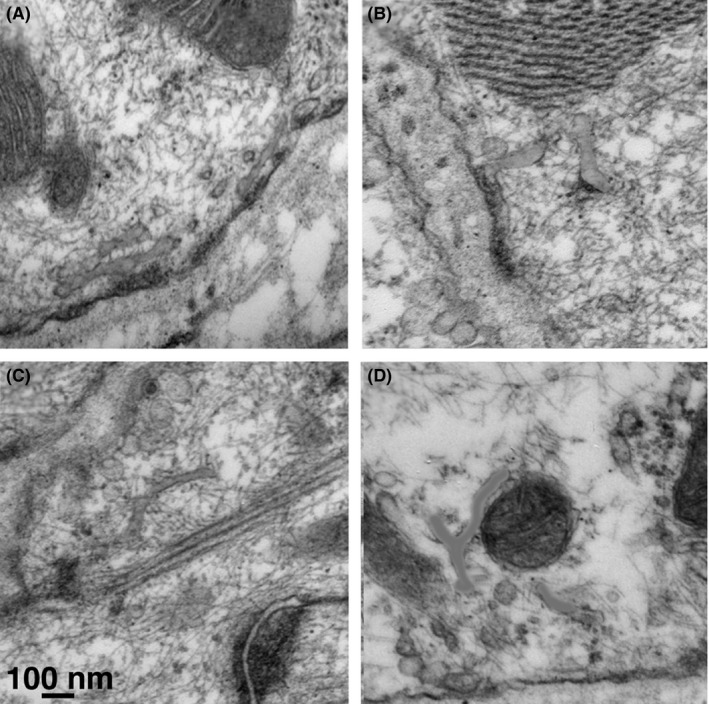
Images of SAN cells showing free SR in four different cells (A‐D), here highlighted in orange, where the Ca^2+^ pumping ATPase is located. The free SR elements are located mostly within a peripheral ~0.4 µm‐wide rim of the cell

The amount of free SR (fSR), often seen associated with PCs, is quite limited in the cells that we have studied. fSR outlines are only seen in some cell images, and, where visible, SR tubules are mostly limited to the cell periphery (Figure 8). The measured distance between the plasmalemma and the furthest SR element varies between 0.2 and 0.9 µm and on the average free SR profiles lie in a band which is within 0.4 ± 0.2 µm from the plasmalemma (from 30 measurements).

To obtain a value for the volumes of junctional SR (in PCs) and of free SR in the cells, we measured the surface areas of the sectioned outline of the two elements and compared it to the surface areas of the sectioned outline of the cells. The ratio between areas of PCs and fSR area and the cell area is the same as the volume ratios of the two organelles. The average area of sectioned PCs, calculated from the measured average length and width of 29 PCs is 0.0043 ± 0.0012 µm^2^. The average number of PCs/cell was 5.3 ± 1.2 and the average area of sectioned cell profile was 73.11 ± 17.82 µm^2^. From this, we calculate the percentage of sectioned cell area occupied by jSR area to be 0.03 (see Table 1, column 2). The average free SR area in the same cell profiles was 0.03 ± 0.04 µm^2^, and using the above data for average area of cell profile, the calculated percentage of sectioned cell area occupied by fSR is 0.04 (Table 1, column3). Outlines of the total SR (jSR + fSR) occupy 0.07% of the cell outline (Table 1, column 4).

**Table 1:** Morphometric parameters of rabbit SAN cells compared with other cardiac myocytes^22^, ^24^, ^35^, ^36^

Tissue	Maximum fSR to sarcolemma distance, µm (2/30/30)^e^	fSR volume/ cell volume, % (2/29/94)^e^	# of PC/cell perimeter µm (2/29/29)^e^	jSR volume/ cell volume, % (2/29/133)^e^	Total SR volume/ cell volume, % (calculated)
Rabbit SAN	0.4 ± 0.2	0.04 ± 0.05	0.13 ± 0.03	0.006 ± 0.002	0.05 ± 0.06
Mouse ventricle^c^		0.65		0.22	0.87
Rat ventricle^b^		3.2		0.3	3.5
Rabbit ventricle^a^		2.4		2.2	4.6
Frog sartorius^d^		5		4.1	9.1

Note

Values for Rabbit SAN row are expressed as mean ± standard deviation.

a Brochet et al,^31^ these values were obtained by a less precise morphometric analysis and they refer to “cytoplasmic” volume that is total volume minus mitochondria, nuclei, and SR volumes.

b Page et al, 1971^32^

c Bossen et al, 1978,^33^

d Mobley and Eisenberg, 1975^34^

e (Number of hearts/Number of cells/Number of measurements)

### 3.3 Plasmalemma details: intercellular communication and caveolae

Cells within the sinus node are tightly packed and constrained in close proximity with each other (Figure 2), so they have multiple interactions (Figures 3, 4, 5 and Figure S1). Pale and dark cells are randomly mixed and their relationships to each other involve three configurations. Some part of the cell, for example, the upper surface and part of the left side in Figure 4, is separated from the neighboring cells by a double layer of basal lamina; in other regions, for example, the lower part of Figure 4, at left the basal laminae of the two adjacent cells are fused into one; the rest of the cell surface is involved in a prolonged region of close contacts with one of its neighbors. Several densities on the cell surface are hemi‐adhering junctions that allow anchorage to the extracellular network, via the basal lamina. At some sites, a direct mechanical connection with the neighboring cell is established via adhering junctions. Close appositions of naked membranes are quite frequent and are not present in the working myocardium except at intercalated discs. The average percentage of cell surface involved in the close proximity with other cells without an intervening basal lamina is 30 ± 16% (from 30 cells) and each sectioned cell profile contacts on average two other cells. Thus, approximately 30% of the cell surface has the potential of establishing a site of communication with adjacent cells.

**Figure 9:**
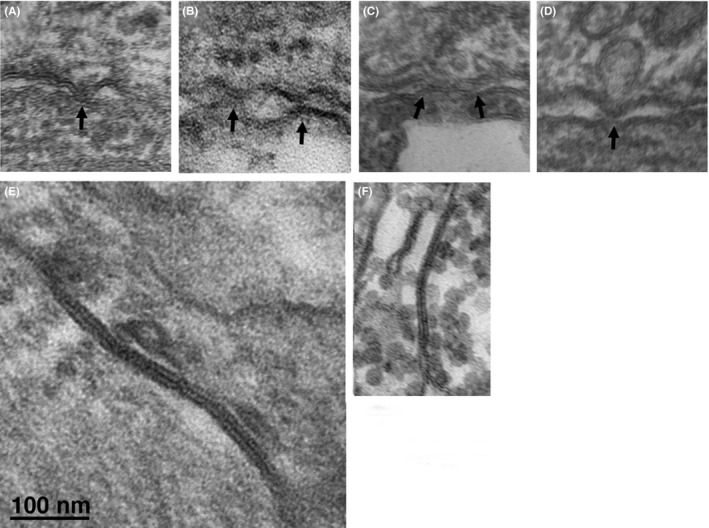
SAN cells are not joined by complex intercalated discs, but the extensive areas of close proximity between the cells offer the opportunity for location of communication sites. (A‐D) Arrows indicate sites of small contacts between cells, presumably mini gap junctions. (E) Classical image of an extensive gap junction between two intermediate cells. Such classical gap junctions are extremely rare between primary pacemakers cells, but one is shown in (F)

We surveyed extensive areas of cell contacts within the sinus node and, with very few exceptions, we found no evidence for small but identifiable gap junctions. In the search for possible cell contacts, we encountered only a single small recognizable, classical gap junction with closely apposed membranes (Figure 9F), similar to a larger junction between cells of either intermediate type of invading atrial cells (Figure 9E). However, a further close look at images from contact regions in very thin sections of cells from the inner core that had been treated to enhance contrast revealed small punctate junctions (Figure 9A‐D) of the type described by Masson‐Pevet et al and by Irisawa.^17^, ^19^ Unfortunately, a realistic estimate of their frequency is not possible due to the difficulty in visualizing them.

**Figure 10:**
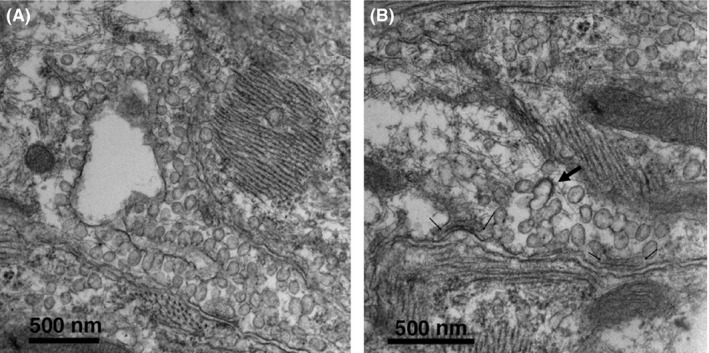
Images from two different cells. Caveolae, each appearing as a small‐membrane‐limited balloon are found in extensive clusters, as illustrated here, that are unevenly distributed over some parts of the cell surfaces. We found no clue to the reason for this uneven distribution. Caveolae are not usually associated with active endocytic processes, but a coated endocytic vesicle is rarely associated with a multiple caveolar invagination (arrow in B). Small arrows indicate peripheral couplings

The plasmalemma of primary pacemaker cells is richly endowed in caveolae (Figure 10A,B). However, the distribution of caveolae is uneven, since many cell outlines are practically devoid of them (eg, see Figures 4 and 5).

## 4 DISCUSSION

One major question in the functioning of the primary pacemaking cells is whether or by how much a “calcium clock” may be involved in determining their periodic action‐potential activity.^23^ Previous model projections on the magnitude of the calcium clock events^9^, ^10^ were based on quantitative data for SR content of the ventricular myocardium. On that basis, a well‐defined calcium wave could be suggested to be at the basis of the observed calcium signals in isolated cells. We find, however, that both free and junctional SR volumes of cells strictly identified from their location in the pacemaking center of the node are a small fraction of that found in ventricular myocardium, in the rabbit (Table 1). Additionally, the free SR is restricted to a peripheral band within the pacemaking cells, and in agreement with Musa et al,^25^ there are neither T tubules nor corbular SR. Keeping in mind the well‐characterized identity of the cells described here, it will be of primary importance to determine how these data, particularly the scarcity of calcium pumping SR, affect calculations of calcium wave activity.^26^ This work originated from the specific requirement for quantitative data (mostly extent and distribution of SR components) necessary for answering the above questions. Therefore, our methods were limited to electron microscopy.

The primary pacemaking cells in the core of the rabbit SAN connect to each other at their ends via adhering junctions, of the type present at intercalated discs of the working myocardium and face each other at their lateral borders across extensive narrow gaps that occupy ~30% on the average of their total surface. In the past, close examinations of the cell surfaces by electron microscopy and following the use of antibodies failed to reveal either the structural signature of gap junctions or aggregates of CX43 and CX45 (two cardiac specific connexins), see introduction. Our close examination of the core peacemaker cells confirms that classical aggregates of connexons are extremely rare in the inner core of the rabbit SAN, but that “mini” junctions of the type described by Irisawa^19^ are present. A few connexons located at such small contact sites would probably be sufficient for electrotonic transmission coordinating the pacemaking events and would protect the cells from unwanted backfiring.^27^ An alternative hypothesis that has gained ground in recent years is the concept that electric fields and/or extracellular accumulation of ions generated by action in one cell may modulate current flowing through channels in a neighboring cell, constituting communication by ephaptic transmission.^28^ However, such transmission requires specific anatomical basis, such as the creation of restricted spaces^29^ and there is no evidence that such spaces are present at the extensive lateral appositions of pacemaking cells.

## 5 LIMITATIONS

The limitations of this study include small sample size and the fact that only male rabbits were included for analysis. Gender‐based morphological differences in rabbit sinus node have not been described in the literature and to keep the sample homogenous only male rabbits were included for the study.

## CONFLICT OF INTEREST

The authors have no conflicts of interest.

## AUTHOR CONTRIBUTIONS

Ramesh Iyer, Clara Franzini‐Armstrong, and Oliver Monfredi designed and performed microscopy, wrote paper; Manuela Lavorato and Mark Terasaki performed and analyzed 3D reconstruction by serial sectioning and SEM.
