# Supplementary figures and images for: Ultrastructure of primary pacemaking cells in rabbit sino‐atrial node cells indicates limited sarcoplasmic reticulum content
Source: FASEB Bioadv. 2020 Jan 7;2(2):106–15. doi: 10.1096/fba.2018-00079 (PMC7003656; doi:10.1096/fba.2018-00079)

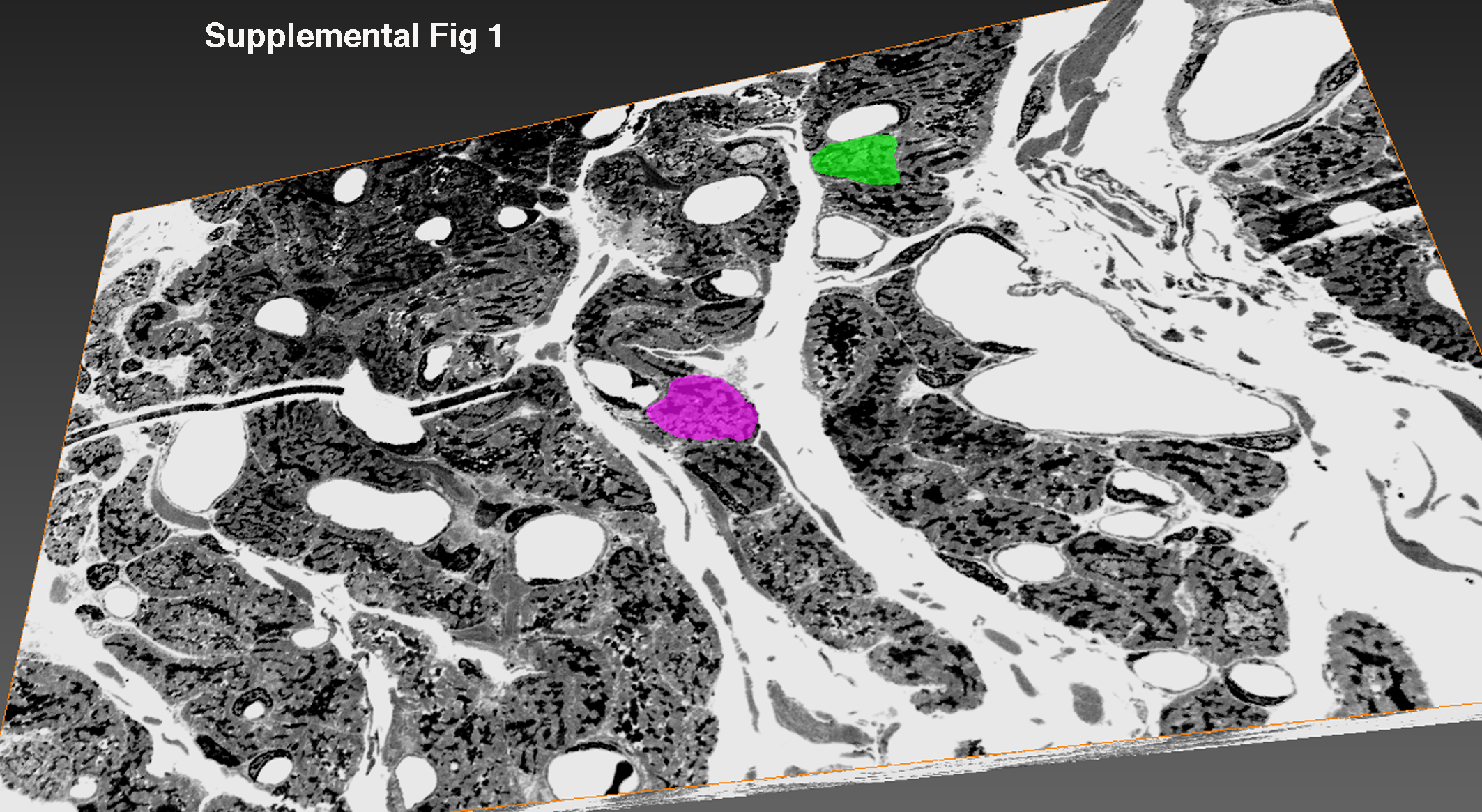

Supplement: Supplementary file 1 [file FBA2-2-106-s001.tif]

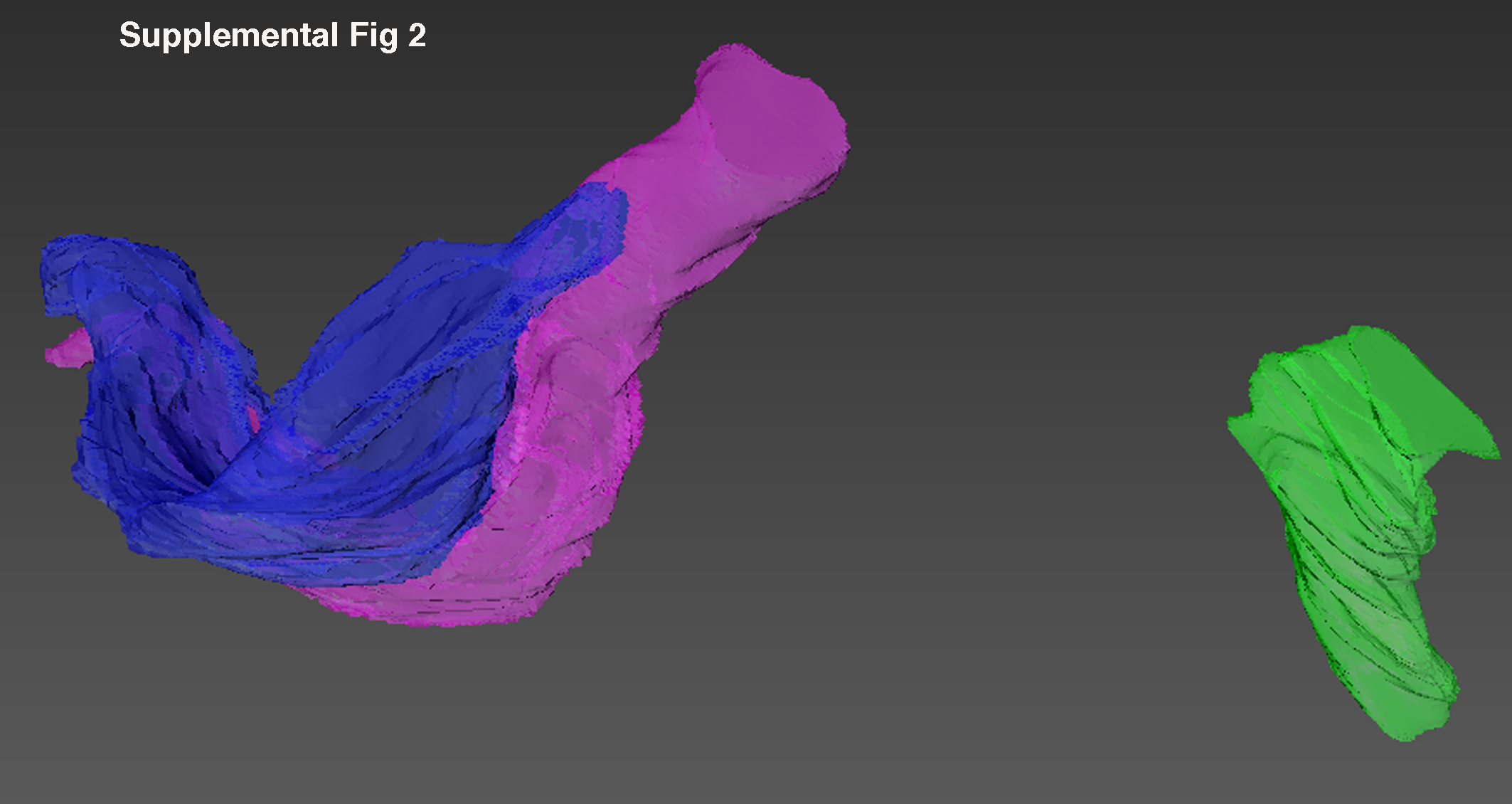

Supplement: Supplementary file 2 [file FBA2-2-106-s002.tif]
