# Supplementary material for: Ultrastructure of primary pacemaking cells in rabbit sino‐atrial node cells indicates limited sarcoplasmic reticulum content
Source: FASEB Bioadv. 2020 Jan 7;2(2):106–15. doi: 10.1096/fba.2018-00079 (PMC7003656; doi:10.1096/fba.2018-00079)
Supplement: Supplementary file 3 [file FBA2-2-106-s003.docx]

**Supplemental Methods**

For 3-D imaging of SAN cells’ outlines, a series of 500 nm thick sections were obtained in a ATUMtome ultramicrotome (Boeckeler Instruments, Tucson, Arizona) from one of the tissue blocks previously used for thin sectioning. The sections were automatically mounted on tape for viewing by scanning microscopy (35) and imaged with a Verios field emission electron microscope. (FEI, Hillsboro, OR) in backscatter mode (5 keV electrons, 0.4 nA beam current). The mapping and automated image collection was accomplished using the MATLAB program SEM Navigator (kindly provided by Daniel Berger, Harvard University to M. Terasaki) with some custom software modifications (36). Images were aligned using the Linear Alignment with SIFT algorithm of FIJI. ImageJ (https://fiji.sc/). A series of sections showing no folding was used to reconstruct the shape of two adjacent cells (Fig. S1). Tomograms were obtained using IMOD (version 4.7) (IMOD software package, University of Denver, Denver, CO). A Video (Movie S1) were created using series of snapshots made in IMOD and importing those images into a movie-making software, Videomach (from www.gromada.com). Cell surface segmentation was obtained by outlining the profile in AMIRA 5.6 3D software (FEI). The volumes were reconstructed using direct volume rendering of 3D images with shadings (volren). Two adjacent cells are shown in Fig. S2.

**Supplemental Figure Legends**

Supplemental figure S1. SEM image of the first in a serial section used to reconstruct the 3D outline of 3 SAN cells (see movie). Two initial cell outlines are segmented: the one outlined in purple is further illustrated in Figure S2 together with its immediate neighbor.

Supplemental figure S2. 3D view of two cell outlines from the primary node regions illustrated in Fig. S1. Note the convoluted shapes of the cells and the very extensive areas of surface proximity.

Supplemental Movie S1. Illustrates the segmentation and reconstruction of the two cells in Figure S2.
